# Prognostic Significance of Metabolic Parameters and Textural Features on ^18^F-FDG PET/CT in Invasive Ductal Carcinoma of Breast

**DOI:** 10.1038/s41598-019-46813-5

**Published:** 2019-07-29

**Authors:** Chin-Chuan Chang, Chao-Jung Chen, Wen-Ling Hsu, Shu-Min Chang, Ying-Fong Huang, Yu-Chang Tyan

**Affiliations:** 1 Department of Nuclear Medicine, Kaohsiung Medical University Hospital, Kaohsiung, Taiwan; 2 Graduate Institute of Clinical Medicine, Kaohsiung Medical University, Kaohsiung, Taiwan; 3 Center for Cancer Research, Kaohsiung Medical University, Kaohsiung, Taiwan; 4 Neuroscience Research Center, Kaohsiung Medical University, Kaohsiung, Taiwan; 5 Departments of Nuclear Medicine, Yuan’s General Hospital, Kaohsiung, Taiwan; 6 Department of Health Business Administration, Meiho University, Pingtung, Taiwan; 7 Department of Medical Imaging and Radiological Sciences, Kaohsiung Medical University, Kaohsiung, Taiwan; 8 Graduate Institute of Medicine, College of Medicine, Kaohsiung Medical University, Kaohsiung, Taiwan; 9 Institute of Medical Science and Technology, National Sun Yat-sen University, Kaohsiung, Taiwan; 10 Department of Medical Research, Kaohsiung Medical University Hospital, Kaohsiung, Taiwan

**Keywords:** Breast cancer, Epidemiology

## Abstract

To investigate the prognostic significance of metabolic parameters and texture analysis on ^18^F-fluorodeoxyglucose positron emission tomography/computed tomography (FDG PET/CT) in patients with breast invasive ductal carcinoma (IDC), from August 2005 to May 2015, IDC patients who had undergone pre-treatment FDG PET/CT were enrolled. The metabolic parameters, including maximal standardized uptake value of breast tumor (SUVbt) and ipsilateral axillary lymph node (SUVln), metabolic tumor volume (MTVbt) and total lesion glycolysis (TLGbt) of breast tumor, whole-body MTV (MTVwb) and whole-body TLG (TLGwb) were recorded. Nine textural features of tumor (four co-occurrence matrices and five SUV-based statistics) were measured. The prognostic significance of above parameters and clinical factors was assessed by univariate and multivariate analyses. Thirty-five patients were enrolled. Patients with low and high MTVwb had 5-year progression-free survival (PFS) of 81.0 and 14.3% (*p* < 0.0001). The 5-year overall survival for low and high MTVwb was 88.5% and 43.6% (*p* = 0.0005). Multivariate analyses showed MTVwb was an independent prognostic factor for PFS (HR: 8.29, 95% CI: 2.17–31.64, *p* = 0.0020). The SUV, TLG and textural features were not independently predictive. Elevated MTVwb was an independent predictor for shorter PFS in patients with breast IDC.

## Introduction

Breast cancer, accounting for 30% of all new cancer diagnoses in women^1^, is a heterogeneous disease presenting various morphological appearances, behavior, and responses to therapy. Among them, approximate 85% to 90% of invasive carcinomas are ductal in origin. Many factors, including patient-related conditions such as age, menopausal status, tumor size, histological grade, lymph node status, the expression of hormone receptors (such as estrogen and progesterone receptors), and the expression of human epidermal growth factor receptor 2 (c-erbB-2) have been identified affecting a patient’s prognosis^2^. Although there have been improvements in screening techniques, breast cancer is still the second leading cause of cancer mortality in all ages of women, and the first leading cause of death from cancer among women aged 20 to 39 the United States^1^. The use of more predictive prognostic factors is essential to estimate prognosis and to be able to recommend the best possible treatment for each patient.

Positron emission tomography/computed tomography (PET/CT) using F-18 fluorodeoxyglucose (FDG) has been widely used for staging, recurrence detection, and assessment of response to therapy in cases of patients with breast cancer. The standardized uptake value (SUV) representing the degree of FDG uptake is the most widely used semi-quantitative parameter in FDG PET/CT and provides information that contributes to the final prognosis^3,4^. However, SUV does not reflect the total glycolytic activity within the entire tumor mass, which is commonly heterogeneous. The role of volumetric parameters derived from FDG PET/CT, such as the metabolic tumor volume (MTV) and total lesion glycolysis (TLG), has been investigated more recently. These two parameters have been shown to be independent prognostic factors for several cancers such as lung, cervical, ovarian and tonsillar cancers. However, in breast cancer, some studies investigating the prognostic values of MTV and TLG showed inconclusive and contradictory results^5–11^.

Tumor texture analysis in FDG PET/CT is another research aspect that has been garnering more interest. It consists of various methods for mathematically quantifying the spatial distribution of voxel intensities in images^12^, allowing for an objective evaluation of the visible tumor properties including heterogeneity. Although measuring tumor heterogeneity is not simple, using imaging techniques can take into account the whole tumor via a non-invasive procedure^13^. For breast cancer, heterogeneity of the PET-derived quantitative measurement has been advocated as a potential prognostic factor^14^. However, the ability of textural features to aid in characterizing tissues and determining tumor aggressiveness remains unclear.

To our knowledge, only few studies have examined the relationship between all PET image-derived parameters, including SUV, MTV, TLG and texture analysis, and patient outcome^15–17^. Therefore, the purpose of the current study is to investigate the prognostic values of all PET image-derived parameters, including texture analysis, in patients with newly diagnosed breast invasive ductal carcinoma (IDC).

## Materials and Methods

### Patient population

This study was conducted retrospectively to analyze the medical records of patients with breast cancer who were treated in the Department of Surgery or Oncology in Kaohsiung Medical University Hospital. The inclusion dates referred to in the study were between August 2005 and May 2015. The clinical inclusion criteria were as follows: patients who had (a) a pathologically proven diagnosis of breast IDC; (b) received a whole-body FDG PET/CT scan for pre-treatment staging; and (c) tumor samples from the biopsy or surgery evaluated immunohistochemically to examine the presence of estrogen receptor (ER), progesterone receptor (PR), c-erbB-2, p53 and Ki-67 proteins. Exclusion criteria were the patient’s age being under 20 years or the patient having a medical history of previous malignancy. The study design was approved by the Institutional Review Board [KMUHIRB-E(I)-20180009]. Patient consent was waived because all of the clinical data were retrospectively collected via the review of the patient medical charts. However, the written permissions from patients upon admission and the examinations, including FDG PET/CT scan, were required. Patients with stage I to III disease received a standard surgical treatment (partial mastectomy or skin sparing mastectomy with transverse rectus abdominis flap reconstruction, with sentinel lymph node biopsy or axillary lymph node dissection) when clinically feasible. Adjuvant radiation therapy, chemotherapy, hormone therapy (for positive ER or PR expression) and/or target therapy (for positive c-erbB-2 expression) were applied under clinical indication. Patients with stage IV (M1) disease at initial diagnosis received chemotherapy and/or hormonal therapy as main therapeutic choices. Surgical intervention and/or palliative radiation therapy may have been added. The treatment choice was decided via the discussion in the multidisciplinary joint conference of breast cancer in accordance with the patient’s clinical condition. The tumors were staged according to the TNM staging system of the American Joint Committee on Cancer (AJCC), 7^th^ edition. The T stage was measured by breast ultrasound. The N and M status was evaluated by breast ultrasound, chest CT or FDG PET/CT, and was ascertained by further pathological confirmation. The observation period spanned from August 2005 to December 2016. Progression-free survival (PFS) was defined as the time from diagnosis to disease relapse, progression or death. Overall survival (OS) was defined as the time from diagnosis to death from any cause.

### FDG PET/CT acquisition

Fasting for more than 6 hours prior to the FDG PET/CT exam was requested. Blood glucose level was controlled to be less than 150 mg/dl before tracer injection. Patients were asked to lie down comfortably for minimized uptake of skeletal muscles after intravenous injection of F-18 FDG (7 MBq per kilogram) with the 55 ± 5 minutes mean uptake time. With an “arm-up” position, the spiral low dose CT scan with 140 kV, 80 mA and 3.75 mm section thickness was acquired from vertex to mid-thigh. Then the reverse direction emission acquisition (4 minutes/bed position) was conducted. All the FDG PET/CT images were acquired with the Discovery ST 16 PET/CT scanner (GE Medical System, Waukesha, Wisconsin, USA). Using previous CT transmission for attenuation correction, the PET images were reconstructed iteratively (i.e. order subset expectation maximization). The reconstructed images were displayed on the Xeleris Functional Imaging Workstation (GE Medical System, Waukesha, Wisconsin, USA) for interpretation.

### Image analysis

The interpretation of PET/CT images and the measurement of SUV were executed by two nuclear medicine physicians, who had clinical experience for more than eight years, and had been blinded to the clinical outcomes at interpretation. A positive lesion on PET/CT was defined as abnormal FDG uptake, either focally or diffusely, which was incompatible with a physiological normal uptake. Disagreements about the interpretation and definition were resolved through discussions to reach a consensus. On the FDG PET/CT image, a circle of region of interest (ROI) that encompassed the primary lesion was drawn slice by slice, and the maximal standardized uptake value of primary breast tumor (SUVbt) was collected over the entire lesion. The maximal standardized uptake value of lymph node (SUVln) was recorded by placing the ROI over the ipsilateral axillary lymph nodes.

The analysis of PET images for MTV calculations was performed on the OsiriX workstation (OsiriX MD 8.0, Pixmeo Sari, Bernex, Switzerland), with exclusion of urinary, myocardial, and brain FDG uptake. The metabolic tumor volume of breast tumor (MTVbt) was defined as the volume of the hypermetabolic primary lesion with the SUV more than 2.5^18^, and the metabolic tumor volume of whole body (MTVwb) of each patient was defined as the total volume of whole-body hypermetabolic lesions with SUV > 2.5. The total lesion glycolysis of breast tumor (TLGbt) was obtained by multiplying the MTVbt by the corresponding mean SUV. The patients’ total lesion glycolysis of whole body (TLGwb) was determined by the sum of the TLGs of all selected hypermetabolic lesions.

We selected reproducible and repeatable parameters for the textural analysis, including the co-occurrence matrix (contrast, homogeneity, dissimilarity, and second angular moment) and SUV-based statistics (SUV skewness, SUV kurtosis, SUV variance, SUV mean, and SUV entropy) of the primary lesion. These parameters were calculated using the open-source software CGITA^19^.

### Statistical analysis

Categorical data were represented as frequencies (percentages) and the continuous variables were presented as mean (standard deviation). The correlations between clinical prognostic factors and metabolic parameters on FDG PET/CT images were analyzed using the Spearman’s rank correlation test. The optimal cut-off values for variables were determined by receiver-operating characteristics (ROC) curves analysis. Using the Kaplan-Meier analysis and log-rank test, the survival curves and difference were obtained in the groups dichotomized by the optimal cut-off values of the metabolic parameters and texture analysis. The impact of every metabolic and clinical parameter on survival was assessed by the univariate and multivariate analyses via Cox proportional hazard model. The statistical analyses were performed with MedCalc Statistical Software version 18.2.1 (MedCalc Software bvba, Ostend, Belgium; http://www.medcalc.org; 2018). A two-tailed *p* < 0.05 was considered statistically significant.

## Results

### Patient characteristics

Table 1 presents the demographic and tumor characteristics of total 35 patients who met the inclusion criteria. Their mean age at diagnosis was 52.2 ± 9.7 years with a range of 33–73 years. The primary lesions in the right and left breast were 48.6% and 51.4% respectively. The mean primary tumor size at diagnosis was 30.8 ± 24.4 mm with a range of 5–120 mm. The majority of patients had grade 2 tumors, had tested positive for nodal metastasis (N+), and were without distant metastasis (M0). Seventeen (48.6%) patients were early-staged (stage I or II), while the other 18 patients (51.4%) were late-staged (III or IV). The positive rate for ER, PR, c-erbB-2 and p53 proteins were 74.3%, 57.1%, 40.0%, and 60.0% respectively. Surgery was performed in 29 patients, among them five patients who had neoadjuvant chemotherapy. There were 14 patients who received partial mastectomy, 15 patients received skin sparing mastectomy with transverse rectus abdominis flap reconstruction. Adjuvant radiation, chemo-, hormonal and/or target therapy was added according to clinical indication. The regimen of chemotherapy included 5-fluorouracil (5-FU), epirubicin, cyclophosphamide, taxotere and navelbine. Hormonal therapy included tamoxifen, letrozole, anastrozole, toremifene and exemestane. Target therapy included trastuzumab, pertuzumab and lapatinib.

**Table 1:** Characteristics at diagnosis of all 35 patients with breast cancer.

Variable	
Age at diagnosis, years	
Range	33–73
mean (SD)	52.2 (9.7)
Laterality, n (%)	
Right	17 (48.6)
Left	18 (51.4)
Histological grade, n (%)	
1	4 (11.4)
2	23 (65.7)
3	8 (22.9)
Tumor status, n (%)	
T1	16 (45.7)
T2	9 (25.7)
T3	5 (14.3)
T4	5 (14.3)
Lymph node status, n (%)	
N0	17 (48.6)
N1	8 (22.9)
N2	5 (14.3)
N3	5 (14.3)
Metastatic status, n (%)	
M0	25 (71.4)
M1	10 (28.6)
Clinical stage, n (%)	
I	10 (28.6)
II	7 (20.0)
III	8 (22.9)
IV	10 (28.6)
ER, n (%)	
Positive	26 (74.3)
Negative	9 (25.7)
PR, n (%)	
Positive	20 (57.1)
Negative	15 (42.9)
C-erbB-2, n (%)	
Positive	14 (40.0)
Negative	21 (60.0)
p53, n (%)	
Positive	21 (60.0)
Negative	14 (40.0)
Ki-67 score, n (%)	
High (≥25%)	18 (51.4)
Low (<25%)	17 (48.6)

SD: standard deviation.

ER: estrogen receptor.

PR: progesterone receptor.

c-erbB-2: human epidermal growth factor receptor 2.

The imaging parameters acquired via pre-treatment FDG PET/CT scans were recorded (Table 2). The mean values of maximal SUVbt and SUVln were 4.77 ± 3.32 and 3.97 ± 3.93 respectively. The mean values of MTVbt/TLGbt and MTVwb/TLGwb were 93.72 ± 228.72/306.72 ± 1111.20 cm^3^ and 143.28 ± 298.40/632.85 ± 1390.50 cm^3^ respectively. The texture analyses of breast tumor were also evaluated. Using the gray-level co-occurrence matrix, the mean values of contrast, homogeneity, dissimilarity and second angular moment were found to be 412620.00 ± 740762.70, 1739.08 ± 4561.58, 36661.60 ± 86021.35, and 498037.11 ± 2187052.42 respectively. Among the SUV-based variables of the primary lesion, the SUV skewness, SUV kurtosis, SUV variance, SUV mean and SUV entropy were 1.51 ± 0.99, 6.17 ± 5.24, 1.29 ± 2.19, 2.01 ± 0.95, and 3.44 ± 0.34 respectively.

**Table 2:** Baseline metabolic parameters and texture analysis of lesions on the pretreatment FDG PET/CT in 35 patients with breast cancer.

	Variable	Mean (SD)
Metabolic parameters	Maximal SUVbt	4.77 (3.32)
	Maximal SUVln	3.97 (3.93)
	MTVbt (cm^3^)	93.72 (228.72)
	MTVwb (cm^3^)	143.28 (298.40)
	TLGbt (cm^3^)	306.72 (1111.20)
	TLGwb (cm^3^)	632.85 (1390.50)
Cooccurrence	Contrast	412620.00 (740762.70)
	Homogeneity	1739.08 (4561.58)
	Dissimilarity	36661.60 (86021.35)
	Second angular moment	498037.11 (2187052.42)
SUV statistics	SUVbt skewness	1.51 (0.99)
	SUVbt kurtosis	6.17 (5.24)
	SUVbt variance	1.29 (2.19)
	SUVbt mean	2.01 (0.95)
	SUVbt entropy	3.44 (0.34)

SD: standard deviation.

SUVbt: standardized uptake value of breast tumor.

SUVln: standardized uptake value of axillary lymph node.

MTVbt: metabolic tumor volume of breast tumor.

MTVwb: metabolic tumor volume of whole body lesions.

TLGbt: total lesion glycolysis of breast tumor.

TLGwb: total lesion glycolysis of whole body lesions.

### Correlation between metabolic parameters, texture analysis, and clinical prognostic parameters

Correlations between the clinical prognostic parameters and the metabolic parameters and textural features from FDG PET/CT scans are listed in Table 3. Using Spearman’s correlation test, the maximal SUVbt was found to positively and significantly correlate with the tumor grade and tumor size. It was also observed that the maximal SUVln was positively and significantly correlated with the N status, M status, and clinical stage. The MTVwb was positively and significantly correlated with tumor size (*p* = 0.0017), N status (*p* < 0.0001), M status (*p* = 0.0006), and clinical stage (*p* < 0.0001). Similarly, the TLGwb was positively and significantly correlated with tumor size (*p* = 0.0012), N status (*p* < 0.0001), M status (*p* = 0.0008), and clinical stage (*p* < 0.0001). Tumors with significantly less differentiation had larger values of contrast (*p* = 0.0822), homogeneity (*p* = 0.0005), dissimilarity (*p* = 0.0017), second angular moment (*p = *0.0007), SUV skewness (*p* = 0.0421), and SUV kurtosis (*p* = 0.0034). Larger tumor size and N status also positively correlated with the value of contrast, homogeneity, dissimilarity, second angular moment, SUV skewness, SUV kurtosis, SUV variance and SUV mean. Among the expression of prognosis-related proteins, the positivity of c-erbB-2 expression correlated significantly with the value of SUV entropy (*r* = −0.342, *p* = 0.0446).

**Table 3:** Correlations between metabolic parameters from FDG PET/CT scans and clinical prognostic parameters.

	Grade	Tumor size	N status	M status	Stage	ER	PR	c-erbB-2	p53	Ki-67
	*p*	*p*	*p*	*p*	*p*	*p*	*p*	*p*	*p*	*p*
Maximal SUVbt	0.0262*	0.0012*	NS	NS	NS	NS	NS	NS	NS	NS
Maximal SUVln	NS	NS	<0.0001*	0.0005*	<0.0001*	NS	NS	NS	NS	NS
MTVbt	0.0003*	0.0332*	NS	NS	NS	NS	NS	NS	NS	NS
MTVwb	NS	0.0017*	<0.0001*	0.0006*	<0.0001*	NS	NS	NS	NS	NS
TLGbt	0.0004*	0.0047*	NS	NS	0.0466*	NS	NS	NS	NS	NS
TLGwb	NS	0.0012*	<0.0001*	0.0008*	<0.0001*	NS	NS	NS	NS	NS
Contrast	NS	NS	NS	NS	NS	NS	NS	NS	NS	NS
Homogeneity	0.0005*	0.0382*	NS	NS	NS	NS	NS	NS	NS	NS
Dissimilarity	0.0017*	0.0225*	NS	NS	NS	NS	NS	NS	NS	NS
Second angular moment	0.0007*	0.0335*	NS	NS	NS	NS	NS	NS	NS	NS
SUV skewness	0.0421*	NS	NS	NS	NS	NS	NS	NS	NS	NS
SUV kurtosis	0.0034*	NS	NS	NS	NS	NS	NS	NS	NS	NS
SUV variance	NS	0.0015*	NS	NS	NS	NS	NS	NS	NS	NS
SUV mean	NS	0.0027*	NS	NS	NS	NS	NS	NS	NS	NS
SUV entropy	NS	NS	NS	NS	NS	NS	NS	0.0446*	NS	NS

*Statistically significant.

NS: not statistically significant.

SUVbt: standardized uptake value of breast tumor.

SUVln: standardized uptake value of axillary lymph node.

MTVbt: metabolic tumor volume of breast tumor.

MTVwb: metabolic tumor volume of whole body lesions.

TLGbt: total lesion glycolysis of breast tumor.

TLGwb: total lesion glycolysis of whole body lesions.

ER: estrogen receptor.

PR: progesterone receptor.

c-erbB-2: human epidermal growth factor receptor 2.

### Identifying the most discriminative cut-off values

Using the ROC curve analysis, the ideal cut-off values for categorizing metabolic parameters and texture analysis into high and low levels were identified (Table 4). The MTVwb contrast and dissimilarity could best distinguish patients into better and worse PFS (all *p* < 0.0001). For OS, the estimated AUCs of MTVwb and TLGwb were 0.88 (*p* < 0.0001) and 0.87 (*p* < 0.0001) respectively.

**Table 4:** The ideal cut-off values in distinguishing the metabolic parameters and texture analysis into high and low levels according to patient survival using ROC analysis.

	PFS			OS		
	Cut-off value	AUC	*p*	Cut-off value	AUC	*p*
Maximal SUVbt	4.0	0.59	NS	4.0	0.59	NS
Maximal SUVln	1.3	0.67	NS	1.3	0.73	0.0232*
MTVbt	35.6	0.79	0.0001*	35.9	0.75	0.0053*
MTVwb	115.4	0.84	<0.0001*	44.3	0.88	<0.0001*
TLGbt	205.9	0.77	0.0010*	158.4	0.73	0.0150*
TLGwb	232.7	0.83	0.0001*	205.9	0.87	<0.0001*
Contrast	185190	0.83	<0.0001*	210636	0.76	0.0012*
Homogeneity	268.7	0.76	0.0021*	404.1	0.72	0.0203*
Dissimilarity	15958	0.83	<0.0001*	27510	0.78	0.0006*
Second angular moment	20654	0.73	0.0079*	10263	0.69	NS
SUV skewness	1.20	0.60	NS	1.50	0.54	NS
SUV kurtosis	2.82	0.66	NS	2.82	0.64	NS
SUV variance	0.50	0.55	NS	0.08	0.53	NS
SUV mean	1.89	0.61	NS	1.94	0.57	NS
SUV entropy	3.51	0.62	NS	3.51	0.57	NS

*Statistically significant.

NS: not statistically significant.

PFS: progression free survival.

OS: overall survival.

AUC: area under the receiver operating characteristic curve.

SUVbt: standardized uptake value of breast tumor.

SUVln: standardized uptake value of axillary lymph node.

MTVbt: metabolic tumor volume of breast tumor.

MTVwb: metabolic tumor volume of whole body lesions.

TLGbt: total lesion glycolysis of breast tumor.

TLGwb: total lesion glycolysis of whole body lesions.

### Patient outcomes according to cut-off values of MTVwb and TLGwb

**Figure 1:**
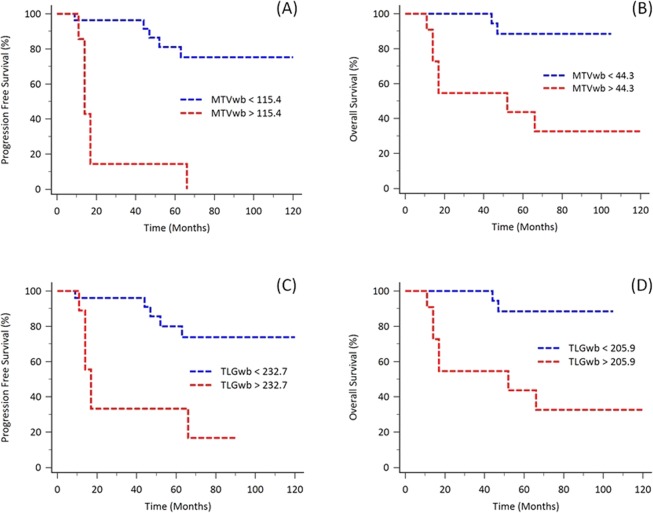
Kaplan-Meier analysis for evaluating the PFS and OS based on MTVwb and TLGwb with the most discriminative cut-off values. Patients with higher MTVwb had significantly shorter survival, compared to those with lower MTVwb (PFS, *p* < 0.0001; OS, *p* = 0.0005; **A**,**B**). Patients with higher TLGwb also had significantly poor outcomes compared to those with lower TLGwb (PFS, *p* = 0.0003; OS, *p* = 0.0005; **C**,**D**).

Using the Kaplan-Meier analysis, it was observed that patients with high MTVwb had shorter clinical survival in comparison to those with low MTVwb levels (PFS, 115.4 cm^3^ as cut-off value, *p* < 0.0001, Fig. 1A; OS, 44.3 cm^3^ as cut-off value, *p* = 0.0005, Fig. 1B). The 5-year PFS for high MTVwb (n = 8) and low MTVwb (n = 27) patients were 14.3% and 81.0% respectively. The 5-year OS for high MTVwb (n = 12) and low MTVwb (n = 23) were 43.6% and 88.5% respectively. Patients with higher MTVwb (≥44.3 cm^3^, n = 12) had the median OS time for 52.0 months (95% CI: 14.0–66.0).

Similarly, patients with high TLGwb had shorter clinical outcomes, compared to those with low TLGwb levels (PFS, 232.7 cm^3^ as cut-off value, *p* = 0.0003, Fig. 1C; OS, 205.9 cm^3^ as cut-off value, *p* = 0.0005, Fig. 1D). The 5-year PFS for high TLGwb (n = 10) and low TLGwb (n = 25) patients were 33.3% and 79.9% respectively. The 5-year OS for high TLGwb (n = 12) and low TLGwb (n = 23) were 43.6% and 88.5% respectively. Patients with higher TLGwb (≥205.9 cm^3^, n = 12) had the median OS time for 52.0 months (95% CI: 14.0–66.0).

### Clinical outcomes in patients with different subgroups

We divided patients into early-staged (staged I and II, n = 17) and late-staged (III and IV, n = 18) groups. Among the early-staged patients, higher MTVbt (35.6 cm^3^ as cut-off value, log-rank *p* = 0.0059), TLGbt (48.0 cm^3^ as cut-off value, log-rank *p* = 0.0251), contrast (185190 as cut-off value, log-rank *p* = 0.0149), and dissimilarity (18168 as cut-off value, log-rank *p* = 0.0037) had shorter PFS. Among the late-staged patients, higher MTVwb or TLGwb had shorter clinical PFS and OS. The significant differences of survival were shown in the evaluation of PFS using the dichotomized total MTVwb (115.4 cm^3^ as cut-off value, log-rank *p* = 0.0007) and TLGwb (586.9 cm^3^ as cut-off value, log-rank *p* = 0.0077).

According to tumor grading, patients with less differentiation of the primary tumor had shorter survival, however, not statistically significant (log-rank *p* = 0.1762 for PFS and *p* = 0.1962 for OS). As to the receptor status, there were no statistically significant difference regarding PFS and OS whether patient was expressing ER, PR and C-erbB-2 or not.

### Univariate and multivariate analysis for clinical impacts of prognostic parameters in all patients

We used the Cox proportional hazard model to evaluate the impact of metabolic parameters, values of texture analysis, and clinical parameters on patients’ clinical outcomes. The best discriminative cut-off values of metabolic parameters and texture analysis were dichotomized by the ROC curve analysis, as mentioned in the previous paragraph. For PFS, the univariate analysis revealed that a larger tumor size (≥3.5 cm, *p* = 0.0062), positive N status (*p* = 0.0188), greater lymph node involvement (≥5*, p* = 0.0332), positive M status (*p* = 0.0275), higher clinical stage (*p* = 0.0053), higher maximal SUVbt (≥4.0, *p* = 0.0467), higher MTVbt (≥35.6 cm^3^, *p* = 0.0091), higher MTVwb (≥115.4 cm^3^, *p* = 0.0001), higher TLGbt (≥205.9 cm^3^, *p* = 0.0131), higher TLGwb (≥232.7 cm^3^, *p* = 0.0017), higher contrast (≥185190, *p* = 0.0001), higher homogeneity (≥268.7, *p* = 0.0334), higher dissimilarity (≥15958, *p* = 0.0100), higher second angular moment (≥20654, *p* = 0.0251), higher SUV mean (≥1.89, *p* = 0.0260) were significantly correlated with shorter clinical outcomes (Table 5). Further, multivariate analysis disclosed that a higher MTVwb [hazard ratio (HR): 8.29, 95% confidence interval (CI): 2.17–31.64, *p* = 0.0020] had the only independent clinical impact on PFS.

**Table 5:** Cox proportional hazards models analysis of potential prognostic factors affecting PFS.

	Univariate analysis		Multivariate analysis	
	HR (95% CI)	*p*	HR (95% CI)	*p*
Age (<vs. ≥50 years)	0.76 (0.28–2.52)	0.7641		
Laterality (Right vs. Left)	1.18 (0.39–3.54)	0.7655		
Differentiation	2.24 (0.85–5.90)	0.1038		
Tumor size (≥vs.<3.5 cm)	5.59 (1.63–19.16)	0.0062*		
N status (positive vs. negative)	3.62 (1.14–11.46)	0.0188*		
Number of LN (≥vs.<5)	3.50 (1.10–11.05)	0.0332*		
M status (+ vs.−)	3.65 (1.15–11.56)	0.0275*		
Clinical stage	2.12 (1.19–3.77)	0.0053*		
ER (positive vs. negative)	0.50 (0.16–1.54)	0.2268		
PR (positive vs. negative)	0.43 (0.14–1.32)	0.1392		
c-erbB-2 (positive vs. negative)	1.64 (0.55–4.92)	0.3708		
p53 (positive vs. negative)	0.40 (0.11–1.41)	0.1529		
Ki-67 (≥vs.<25%)	2.48 (0.26–24.04)	0.4335		
Maximal SUVbt (≥vs.<4.0)	3.06 (0.99–9.45)	0.0467*		
Maximal SUVln (≥vs.<1.3)	3.23 (0.88–11.83)	0.0765		
MTVbt (≥vs.<35.6 cm^3^)	7.50 (1.65–34.07)	0.0091*		
MTVwb (≥vs.<115.4 cm^3^)	11.77 (3.54–39.1)	0.0001*	8.29 (2.17–31.64)	0.0020*
TLGbt (≥vs.<205.9 cm^3^)	4.02 (1.34–12.07)	0.0131*		
TLGwb (≥vs.<232.7 cm^3^)	6.60 (2.04–21.37)	0.0017*		
Contrast (≥vs.<185190)	5.04 (1.59–12.15)	0.0001*		
Homogeneity (≥vs.<268.7)	9.22 (1.19–71.29)	0.0334*		
Dissimilarity (≥vs.<15958)	14.80 (1.9–115.1)	0.0100*		
Second angular moment (≥vs.<20654)	3.60 (1.17–11.07)	0.0251*		
SUVbt skewness (≥vs.<1.20)	0.52 (0.17–1.56)	0.2412		
SUVbt kurtosis (≥vs.<2.82)	5.04 (0.66–38.90)	0.1202		
SUVbt variance (≥vs.<0.50)	2.46 (0.82–7.35)	0.1080		
SUVbt mean (≥vs.<1.89)	3.49 (1.16–10.47)	0.0260*		
SUVbt entropy (≥vs.<3.51)	2.84 (0.87–9.34)	0.0852		

*Statistically significant.

ER: estrogen receptor.

PR: progesterone receptor.

c-erbB-2: human epidermal growth factor receptor 2.

SUVbt: standardized uptake value of breast tumor.

SUVln: standardized uptake value of axillary lymph node.

MTVbt: metabolic tumor volume of breast tumor.

MTVwb: metabolic tumor volume of whole body lesions.

TLGbt: total lesion glycolysis of breast tumor.

TLGwb: total lesion glycolysis of whole body lesions.

HR: hazard ratio; CI: confidence interval.

For OS, the univariate analysis revealed that a larger tumor size (≥3.5 cm, *p* = 0.0119), positive N status (*p* = 0.0279), greater lymph node involvement (≥5*, p* = 0.0404), positive M status (*p* = 0.0031), higher clinical stage (*p* = 0.0035), higher maximal SUVln (≥1.3, *p* = 0.0388), higher MTVwb (≥44.3 cm^3^, *p* = 0.0049), higher TLGbt (≥158.4 cm^3^, *p* = 0.0086), higher TLGwb (≥173.4 cm^3^, *p* = 0.0049), and higher dissimilarity (≥27510, *p* = 0.0199) were significantly correlated with shorter clinical outcomes (Table 6). The multivariate analysis further revealed that a higher clinical stage (HR: 4.22, 95% CI: 1.38–12.92, *p* = 0.0117) had the only independent clinical impact on OS.

**Table 6:** Cox proportional hazards models analysis of potential prognostic factors affecting OS.

	Univariate analysis		Multivariate analysis	
	HR (95% CI)	*p*	HR (95% CI)	*p*
Age (<vs. ≥50 years)	0.42 (0.12–1.52)	0.1889		
Laterality (Right vs. Left)	0.93 (0.27–3.23)	0.9109		
Differentiation	2.23 (0.73–6.79)	0.1580		
Tumor size (≥vs.<3.5 cm)	6.40 (1.51–27.16)	0.0119*		
N status (positive vs. negative)	4.69 (0.99–22.21)	0.0279*		
Number of LN (≥vs.<5)	3.98 (1.06–14.95)	0.0404*		
M status (+ vs.−)	7.54 (1.97–28.83)	0.0031*		
Clinical stage	3.59 (1.52–8.48)	0.0035*	4.22 (1.38–12.92)	0.0117*
ER (positive vs. negative)	0.82 (0.21–3.18)	0.7721		
PR (positive vs. negative)	0.82 (0.24–2.84)	0.7502		
c-erbB-2 (positive vs. negative)	2.11 (0.59–7.49)	0.2489		
p53 (positive vs. negative)	1.00 (0.22–4.51)	0.9981		
Ki-67 (≥vs.<25%)	0.65 (0.04–10.33)	0.7570		
Maximal SUVbt (≥vs.<4.0)	2.44 (0.69–8.67)	0.1682		
Maximal SUVln (≥vs.<1.3)	8.86 (1.12–70.16)	0.0388*		
MTVbt (≥vs.<35.9 cm^3^)	4.57 (0.97–21.58)	0.0549		
MTVwb (≥vs.<44.3 cm^3^)	9.58 (1.98–46.30)	0.0049*		
TLGbt (≥vs.<158.4 cm^3^)	5.52 (1.54–19.73)	0.0086*		
TLGwb (≥vs.<205.9 cm^3^)	9.58 (1.98–46.30)	0.0049*		
Contrast (≥vs.<210636)	9.85 (1.23–66.82)	0.9530		
Homogeneity (≥vs.<404.1)	4.68 (0.99–22.24)	0.0522		
Dissimilarity (≥vs.<27510)	5.01 (1.29–19.45)	0.0199*		
Second angular moment (≥vs.<10263)	3.20 (0.82–12.46)	0.0926		
SUVbt skewness (≥vs.<1.50)	0.57 (0.15–2.23)	0.4203		
SUVbt kurtosis (≥vs.<2.82)	3.42 (0.43–27.03)	0.2431		
SUVbt variance (≥vs.<0.08)	0.62 (0.16–2.42)	0.4883		
SUVbt mean (≥vs.<1.94)	2.85 (0.82–9.88)	0.0990		
SUVbt entropy (≥vs.<3.51)	2.81 (0.72–10.90)	0.1364		

*Statistically significant.

ER: estrogen receptor.

PR: progesterone receptor.

c-erbB-2: human epidermal growth factor receptor 2.

SUVbt: standardized uptake value of breast tumor.

SUVln: standardized uptake value of axillary lymph node.

MTVbt: metabolic tumor volume of breast tumor.

MTVwb: metabolic tumor volume of whole body lesions.

TLGbt: total lesion glycolysis of breast tumor.

TLGwb: total lesion glycolysis of whole body lesions.

HR: hazard ratio; CI: confidence interval.

## Discussion

In the present study, we evaluated the relationships and prognostic values of clinical, pathological, and PET image-derived parameters in patients with newly diagnosed breast cancer. The multivariate analysis showed that higher MTVwb independently affected the PFS. For the evaluation of OS, higher clinical stage was the independent prognostic factor.

Breast cancer is responsible for the second leading cause of women cancer mortality in the developed world^1^. The use of prognostic and predictive factors is essential to estimate prognosis and to be able to recommend the best possible treatment for each patient afflicted with breast cancer. The most significant prognostic factor in breast cancer is the status of lymphatic nodal metastasis^20^. Positive lymphatic nodal metastasis is sometimes coupled with a worse prognosis, and patients often require systemic chemotherapy and more extensive radiotherapy. The expression of ER is considered a predictive and good prognostic marker for endocrine treatment^21^. During the first 5 years after diagnosis, the patient with a higher level of ER is often associated with a favorable prognosis and a lower risk of recurrence and death from breast cancer. However, the prognostic value shifts and with longer follow-up, ER-positive breast cancer is often associated with late recurrence (beyond 5 years) compared with ER-negative tumors^22–24^. The prognosis value of PR has been shown in several studies, even independent from ER and other prognostic markers^25^. C-erbB-2 is a transmembrane protein functioning as a tyrosine kinase. The over-expression of c-erbB-2 was considered to be associated with higher relapse rate, and subsequently, the mortality rate increased without targeted treatment^26^. And the histological grade, in the case of primary breast cancer, has repeatedly shown to be a strong independent prognostic factor^27–29^.

Since 2008, there have been studies investigating correlations between uptake values on FDG PET/CT scans and clinical prognostic factors in patients with breast cancer^30–33^. Recently, more studies have discussed the relationships between FDG PET/CT image-derived parameters and clinical and pathological factors to aid in treatment planning and determining the prognosis of patients with primary breast cancer; however, they have reported highly variable results. Kaida *et al*.^34^ reported a significant relationship between ER expression and triple negative status, and the TLG, rather than SUVmax and MTV, better reflected the association between tumor metabolism and clinico-pathological factors of breast cancer. Another study conducted by Groheux *et al*.^35^ investigated patients in accordance with three phenotype subgroups (Her-2-positive, triple negative, and ER-positive/Her-2-negative breast cancers). They found that SUVmax and TLG differed among the subtypes and concluded that none of the PET-derived parameters offered high discriminative power in differentiating between the prognostic subtypes of breast cancers. Kajary *et al*.^10^ reported that the SUVmax may reflect tumor metabolism more reliably when compared with the SUVmean, MTV or TLG. Aktas *et al*.^7^ carried out the study to evaluate the relationship of baseline metabolic parameters for the primary tumor with clinico-pathological risk factors and molecular subtypes in patients with invasive ductal breast carcinoma. They found that SUVmax is the most appropriate parameter reflecting immunohistochemical risk factors (molecular subtypes and the Ki-67 index), whereas TLG is mostly associated with clinical risk factors (clinical T size and N stage) and systemic metastasis.

MTV is defined as the volume of tumor tissues with abnormally increased FDG uptake. Studies on gynecological^36,37^, aerodigestive^38,39^, and pulmonary^40^ malignancies as well as lymphoma^41^ have shown that the MTV or TLG are significantly correlated with survival and provide better prognostic value than just the SUV. Some studies have also discussed the relationship between the MTV and prognostic outcomes in patients with breast cancer. Kim *et al*.^8^ concluded that PET indices seem to be useful in the preoperative evaluation of prognosis and that the MTV of lymph nodes and tumor might be considerable factors associated with patient outcome in the context of operable breast cancer. Son *et al*.^9^ evaluated the prognostic value of whole body MTV for patients with metastatic breast cancer, showing that whole body MTV was an independent prognostic index of OS in patients with IDC of the breast with distant metastasis at the time of initial diagnosis. Marinelli *et al*.^42^ assessed the correlation between metabolic tumor burden and OS in patients with metastasized triple negative breast cancer. Their analysis showed that SUVmax and TLG were not significantly predictive of survival, yet MTV was significant. A similar study concluded that MTV may be associated with axillary lymph node status in breast cancer patients, particularly in T2 and T3 stages^6^. Another study by Hyun *et al*. reported that, regardless of tumor subtypes and pathologic tumor response, the volume-based metabolic tumor response to neoadjuvant chemotherapy is associated with the risk of recurrence^5^.

Image texture features have already been described for non-medical applications a few decades ago. A new emerging field “radiomics” that decodes the tumor phenotype with non-invasive imaging procedures has been met with growing interest^43^. Without biopsy, texture features could potentially be used to realize the entire tumor lesion and to predict the response to the treatment and the patient’s outcome. Several previous studies have reported that inhomogeneous FDG uptake is related to the heterogeneity of histopathological features in various malignancies such as non-small cell lung cancer^44^, head and neck squamous cell carcinoma^45^, and oligodendroglioma^46^. However, the causes of the heterogeneous distribution of FDG within a tumor are still not fully understood. It has been reported that, in sarcoma and cervical cancer, the intratumoral heterogeneity of FDG uptake is significantly correlated with patient outcomes^47,48^. This aspect has also been used to tailor therapeutic strategies, including defining the target volume or optimizing the dose distribution in planning for radiotherapy^49,50^. For breast cancer, Soussan *et al*.^14^ mentioned that tumors with heterogeneous textural indices in FDG PET/CT led to poorer prognosis. They suggested that textural analysis might be considered, in addition to SUVmax, as a new tool in assessing tumor aggressiveness. Another study was designed to evaluate the relationships between textural features, metabolic parameters, and tumor characteristics, as well as the capability of those parameters in predicting response to neoadjuvant chemotherapy^51^. Subsequently, a significant association between textural features and the histological type was observed. Additionally, SUVmax and TLG were able to predict the response to neoadjuvant chemotherapy, while textural features failed to do so. Son *et al*.^52^ investigated the correlations between intratumoral metabolic heterogeneity and SUVmax, MTV, TLG, disease stage, and the prognosis. Their result demonstrated that the heterogeneity factor had close correlation with the MTV and was the best prognostic factor in predicting OS in patients with IDC. A similar study concluded that MTV was significant after multivariate analysis, while textural analysis is not of added value when predicting event-free survival in ER-positive/Her-2-negative locally advanced breast cancer patients^15^. Interestingly, our data are aligned with the concept that whole body MTV is the only independent prognostic factor for patient survival after multivariate analysis.

There were some limitations in the current study. First, it used a retrospective study design with a small population of patients. A prospective larger cohort study is needed to validate our result. Second, patients with different staging, ER, PR, and c-erbB-2 expressions received different treatment modalities. This might have also caused bias for PFS and OS. Third, the study ignored the partial volume effect, which may lead to bias when tracer uptake in the small tumors is measured. To correct this bias, partial volume correction (PVC) should be performed. There have been several different PVC schemes introduced for the PET tumor imaging and used in different kinds of malignancies^53–56^. However, only a few investigators added the PVC into the study that surveyed the prognostic value of metabolic parameters on FDG PET^57^. In the future, adding the PVC into study to get a more accurate and comprehensive result is taking into consideration. Finally, some patients had too short follow-up durations, i.e. less than five years, and the recurrence may occur even later. A longer follow-up duration may improve the accuracy when evaluating the PFS and OS.

## Conclusion

The current study indicated that pre-treatment of MTVwb, based on FDG PET/CT images could predict survival in patients with breast cancer. An elevated MTVwb was an independent prognostic factor associated with significantly poor PFS.
